# Teach Me Fishing or Give Me the Fish: Differential Effects of Receiving Autonomous and Dependent Help on Task Performance

**DOI:** 10.3390/ijerph20010647

**Published:** 2022-12-30

**Authors:** Beijing Tan, Ziyi Li, Huan Cheng, Zijing Wang

**Affiliations:** School of Management, Huazhong University of Science and Technology, Wuhan 430074, China

**Keywords:** receiving help, task-focused processes, self-focused processes, task performance, perceived prosocial motivation

## Abstract

Research on workplace helping behavior highlights the need for a more balanced perspective that acknowledges both the positive and negative consequences of receiving help. The purpose of this study is to investigate how the mechanisms through which we receive autonomous and dependent help differentially impact recipient task performance, as well as the boundary condition for such effects. Drawing on social information theory, we examined the mediating role of task- and self-focused processes, and the moderating role of perceived prosocial motivation. Through a two-wave and two-source field survey, we collected matched data from 350 employees and their direct supervisors. We examined our hypothesized model with path analysis using Mplus 7.4. Results indicated that receiving autonomous help improved task performance by leading recipients into task-focused processes, and perceived prosocial motivation further strengthened this positive indirect relationship. In contrast, receiving dependent help reduced task performance by eliciting recipients to engage in self-focused processes, and perceived prosocial motivation further augmented this negative indirect relationship. Overall, we spotlight the differential consequences of receiving autonomous and dependent help on recipients and encourage further inquiry about the role of social information processing in the helping literature.

## 1. Introduction

Helping behavior at work refers to an affiliative and promotive behavior that aims to support recipients with work-related problems [1], and is therefore assumed to facilitate employee task performance [2]. Nevertheless, a number of studies found complex and sometimes even detrimental effects of workplace help on recipients’ performance-related outcomes [3,4,5,6]. Recent studies seek to understand these mixed findings by introducing the notions of autonomous and dependent help and investigating their differential effects [7,8,9,10]. Research in this space primarily considers competence-related perception as an underlying mechanism without examining the processes by which different types of help are translated. This omission has led to disagreement in predicting the effects of receiving autonomous and dependent help. For example, while Lee et al. [7] assumed that both empowering and non-empowering acts (which correspond to autonomous and dependent help, respectively) potentially facilitate recipients’ competence perception by enabling learning; Lin [8] implied that autonomous help enhances self-efficacy by fostering learning, but that dependent help hampers self-efficacy by eliciting social comparison. Therefore, other than merely focusing on competence perception, it is necessary to examine how different types of help are processed when accounting for the effects of autonomous and dependent help on recipients’ task performance.

In this study, we aim to uncover how and when the receipt of autonomous and dependent help is differentially processed based on the social information processing theory [11], and further examine their divergent consequences on recipient task performance. According to social information theory, salient sources of social information from coworkers have influences on shaping subsequent cognitive processing [11]. We specifically looked at helping behaviors provided by coworkers, because helping interactions are most prevalent among coworkers in everyday work experience [12]. Helping behavior conveys information cues about coworkers’ evaluations of the target employee’s past and present performance or capabilities and also expectations for the future [13,14,15]. Such information will lead to various behavioral outcomes by focusing recipients’ attention and processing certain information [11]. By providing necessary tools or hints for recipients to solve a problem independently [16,17], autonomous help conveys developmental information cues that recipients are expected to develop the competence and ability to solve problems independently in the future. Accordingly, the learning opportunities and positive expectations brought about by autonomous help will direct recipients to task-focused processes where recipients focus attention on, and allocate cognitive capacity toward, generating better task strategies [18,19]. On the contrary, dependent help provides a complete solution or directly solves the problem for the recipient [16,17], revealing evaluative information that stresses the recipient’s inferiority [20]. Because such evaluative information is negative and threatening, receiving dependent help will divert recipients to self-focused processes where recipients direct their attention to threatening consequences and worry about their self-image from the viewpoint of others [19,21,22]. We expect that receiving autonomous help will foster task performance by inducing task-focused processes, whereas receiving dependent help will hinder task performance by eliciting self-focused processes.

Because of the discretionary nature of helping behavior [1], receiving help often triggers recipients’ attribution about why help is provided [23]. We further propose that motivation attributed to coworker help places a boundary condition for information processing after receiving help. Prior work suggests that perceived prosocial motivation increases the value of helping behaviors [24,25]. Perceived prosocial motivation reflects the extent to which the recipient perceives that the helper is aiming to benefit and satisfy the recipient’s needs [26]. When coworker help is perceived as originating out of prosocial motivation, recipients may put more weight on it [6] and thus be more likely to recognize or accept the information delivered by that help. We posit that under higher perceived prosocial motivation, receiving autonomous help should engender greater task-focused processes and higher task performance, because recipients will tend to place more value on the developmental information and make better use of the learning opportunities brought about by autonomous help. On the contrary, under higher perceived prosocial motivation, receiving dependent help should engender greater self-focused processes and lower task performance because recipients may give more credence to the negative evaluative information conveyed through dependent help, exacerbating their worries about self-image.

The current study tests these hypotheses in a two-wave and two-source field survey. By demonstrating support for our hypotheses, our research makes three major contributions. First, by introducing task-focused and self-focused process mechanisms, we provide a new lens through which to understand why receiving autonomous help may facilitate, whereas receiving dependent help may hinder, task performance beyond the competence perception mechanism [7,10]. In so doing, we also extend the nascent literature on the downside of coworker help in task performance. Second, to the best of our knowledge, this research is the first to contribute a social information-processing theoretical lens [11] to the literature on autonomous and dependent help. We advance the idea that autonomous help conveys developmental information that directs employees to a mode of processing focused on the tasks at hand, whereas dependent help implies evaluative information that diverts employees to a mode of processing focused on the self. In addition, while previous research primarily stressed the instrumental nature of autonomous and dependent help, our research enriches understanding of the nature of the two types of help by adding an informational aspect. Third, previous research pointed to the importance of recipients’ attribution in influencing the effects of help [6,7]. Extending this line of work, we show perceived prosocial motivation as a contingency, which, together with the autonomous–dependent help distinction, provides a new way to reconcile conflicting views regarding the role of help. In a nutshell, we not only show that informational cues contained in help matter by directing recipients to differential processing modes, but also offer the perceived prosocial motivation of helpers as a boundary condition for this account.

## 2. Theoretical Background and Hypotheses Development

### 2.1. An Overview of Autonomous and Dependent Help at Work

Helping behaviors manifest in a variety of ways at work. In his study on help-seeking behaviors, Nadler [16,17] distinguished between autonomous help and dependent help. Autonomous help refers to providing the recipient with the necessary tools and knowledge to solve problems independently, whereas dependent help involves offering the ready solution or directly taking over the problem for the recipient [16,17]. Autonomous help is suggested to have a more developmental function, as it is often accompanied by more learning elements [16] such as the explanation of fundamental principles underlying the problem [4] and the sharing of new knowledge and skills [27]. In contrast, dependent help has a greater immediate instrumental function because it merely concerns the current problem without teaching the recipient how to tackle similar problems independently in the future [28].

Even though both types of help provide the recipient with resources to assist in task-related problem-solving, previous research has largely demonstrated that autonomous help produces more beneficial effects than dependent help [4,8,9,10,29,30]. Researchers studying workplace autonomous help’s effects on recipients primarily focus on its competence-development function. Autonomous help enables recipients to gain and master relevant knowledge and skills to solve the problem, which should boost recipients’ competence perception and subsequent task performance outcomes [7,9,10]. Previous research also strived to explain the detrimental effects of dependent help on recipients’ performance outcomes through the competence perception mechanism; however, the results were inconsistent. Some scholars found that dependent help negatively impacts recipients’ self-perceived competence [8], while some others found non-significant effects [10] or conditional positive effects [7]. Empirical research on workplace autonomous and dependent help is summarized in Table 1. These research findings imply that competence perception may not adequately explain the mechanisms by which the two types of help affect task performance and, more crucially, that receiving autonomous help and dependent help may impact task performance in varied ways.

### 2.2. Mediating Role of Task and Self-Focused Processes

Drawing from the social information processing theory [11], we propose that receiving autonomous and dependent help may impact recipient performance by directing the recipient to different processing modes. Social information processing theory [11] suggests that social context shapes people’s behavior by structuring their attentional focus, making certain information about the environment more or less salient. In the current study, we introduce task-focused and self-focused processes as mechanisms to capture how coworkers’ help influences task performance. Task-focused processes refer to the processing mechanism where individuals devote a great quantity of cognitive resources to the task with intense focus and concentration [18,19,31]. Self-focused processes refer to the processing mechanism where individuals attend to self-referent information such as one’s behavior, emotion, and cognition [19,21,22,31]. We particularly focus on the public self-focused processes where individuals consider the self as a social object and attend to their self-image from the viewpoint of others [21]. This is because workplace help is delivered in public, or at least can be observed by the helper, and the presence of an audience has been found to elicit self-presentational concerns [32]. Moreover, given that information processing capacity is finite [33], a predominant engagement in task-focused processes should inhabit engagement in self-focused processes and vice versa [19]. We therefore expect receiving autonomous help and dependent help will lead to one predominant processing mode.

Autonomous help provides recipients with learning opportunities to improve their knowledge and skills [27,34] and hence alludes to expectations that recipients are capable of learning how to solve the problem and can solve the problem independently in the future. Although there is no direct evidence that recipients view autonomous help as a sign of helpers’ expectations, prior research suggests that helpers tend to provide autonomous help to those for whom they have high expectations regarding future performance and ability [15]. Accordingly, coworker autonomous help will expose recipients to such developmental expectations, directing recipients to task-focused processes to conform to those expectations. Moreover, receiving autonomous help requires the recipient to participate in the helping interaction [7,35] and invest energy and effort into problem-solving [29], which is more likely to generate heightened levels of task-focused attention. Indeed, empirical research provides suggestive evidence that developmental information cues (e.g., task-involved instructions) [36] foster individuals’ learning regarding how to perform a task effectively.

Dependent help makes recipients reliant on the helpers’ superior knowledge or resources [16,17], which signals a view of the recipient’s inferiority and dependency [20]. Both theoretical and empirical evidence indicates that helpers tend to provide dependent help to those they perceive to be incompetent to effectively solve problems on their own [13,15]. Previous research affirmed the role of the evaluative audience in increasing focus on the public aspects of the self [37], especially when the evaluation is from similar others (e.g., peers who have similar levels of status) [38]. Therefore, dependent help provided by coworkers is likely to divert the recipient’s focus toward the self and toward the threatening consequences of receiving help. For example, recipients will question their competence (e.g., “Am I perceived as incompetent by my coworkers?”) or whether receiving dependent help conveys a negative impression to others (e.g., “How might my coworkers perceive me?”). Indeed, dependent help has been found to be more likely to trigger recipients to feel threatened about their self-image [34,39,40].

To summarize, we propose that receiving autonomous help may focus recipients’ information-processing on mastering and completing the task, whereas receiving dependent help may divert recipients’ focus to how they present in front of others. Thus, we hypothesize:

**Hypothesis** **1** **(H1).** *Receiving autonomous help is positively related to task-focused processes*.

**Hypothesis** **2** **(H2).** *Receiving dependent help is positively related to self-focused processes*.

Task-focused processes have long been deemed important determinants of task performance [18,41,42,43,44,45]. High task performance relies on the extent to which individuals focus attention on, and allocate cognitive capacity for, the detailed processing of the task [41]. When task-focused processes are heightened, individuals may come up with more strategies to improve task performance and invest more effort to overcome difficulties [46], leading to higher task performance. Therefore, by directing recipients to task-focused processes, autonomous help would foster intense concentration and great cognitive resource allocation and, thus, facilitate recipients getting work done effectively.

On the contrary, to the extent that attention is diverted away from the task, performance will decrease accordingly. Because information-processing capacity is limited [33], heightened self-focused processes will make individuals unable to devote sufficient attention to task completion and lower the cognitive resources that should be allocated to the task. Moreover, self-focused processes will lead to recipients’ awareness of their weaknesses, resulting in negative effects such as anxiety, and in turn may drain recipients’ resources to invest in the task [41,47]. Therefore, by engendering self-focused processes, dependent help would prevent recipients from adequately engaging in tasks, resulting in performance decrements.

To summarize, we propose that receiving autonomous help will facilitate task performance through directing recipients to processes focused toward the task, whereas receiving dependent help will hamper task performance through diverting recipients to self-focused processes. Thus, we hypothesize:

**Hypothesis** **3** **(H3).** *Receiving autonomous help has a positive indirect relationship with task performance* via *task-focused processes*.

**Hypothesis** **4** **(H4).** *Receiving dependent help has a negative indirect relationship with task performance* via *self-focused processes*.

### 2.3. Moderating Role of Perceived Prosocial Motivation

Since helping behavior is discretionary [1] and requires the helper to expend extra time and effort performing the helping act [48], it is not surprising that recipients would seek causal explanations for the motivation behind help. In the current study, we specifically focused on recipient-perceived prosocial motivation, which refers to the extent to which recipients view the helper as externally oriented and aimed to benefit and meet the needs of others [26,49]. Prior research mostly highlighted the upside of perceived prosocial motivation for building trust and enhancing social interactions [50,51,52,53]. Relatively less known is whether such attribution can influence the way recipients interpret helping behavior. Recent research suggests that prosocial motivation attribution enhances recipients’ assessments of the usefulness of information [25] and increases the value of helping actions [24,54]. Based on this logic, we expect that when helpers are perceived to be prosocially motivated, information cues conveyed by the helping behavior may be more easily recognized and accepted by the recipient because it is sent by reliable sources.

We propose that perceived prosocial motivation may strengthen the positive relationship between receiving autonomous help and task-focused processes. When helping behavior is attributed to prosocial motivation, recipients are likely to value the efforts of helper and engender fewer doubts regarding the potential benefits of the advice [55,56]. Thus, recipients may place greater value on the learning opportunities provided by the helper and use them to advance their task mastery. Additionally, good intentions may amplify the developmental expectations supplied by autonomous help, as well as develop high levels of task-related competence perceptions and positive beliefs, allowing recipients to concentrate more on their tasks. We further propose that perceived prosocial motivation may also strengthen the positive relationship between receiving dependent help and self-focused processes. Prosocial motivation demonstrates the intention to help based on the assumption about the recipient’s best interests [57], implicitly suggesting that dependent help is the appropriate form for the recipient. As a result, recipients may reconfirm negative competency-related information, creating additional self-doubt, anxiety, or worry. On the other hand, when a lack of capacity is exhibited in a nuanced, active manner, recipients cannot easily ascribe negative information cues to the person giving help [58]; instead, they enhance their own "mental intrusion," such as the inability to repress unwanted thoughts [59,60].

To summarize, we suggest that perceived prosocial motivation is an important boundary condition that influences the recipients’ processing mode after receiving help. Perceived prosocial motivation highlights the developmental information conveyed by autonomous help, thus strengthening the effect of autonomous help on task-focused processes. Meanwhile, perceived prosocial motivation reconfirms the evaluative information signaled through dependent help, leading to aggravated self-focused processes. Thus, we hypothesize:

**Hypothesis** **5** **(H5).** *Perceived prosocial motivation moderates the positive relationship between receiving autonomous help and task-focused processes, such that it is stronger with higher perceived prosocial motivation*.

**Hypothesis** **6** **(H6).** *Perceived prosocial motivation moderates the positive relationship between receiving dependent help and self-focused processes, such that it is stronger with higher perceived prosocial motivation*.

In sum, our theoretical model explains when and why receiving autonomous help may prompt task performance, whereas receiving dependent help may hinder task performance. Overall, our arguments point to the moderation mediation models where the positive indirect relationship between receiving autonomous help and task performance via task-focused processes is stronger with higher perceived prosocial motivation, and the negative indirect relationship between receiving dependent help and task performance via self-focused processes is also stronger with higher perceived prosocial motivation. Taken together, we hypothesize:

**Hypothesis** **7** **(H7).** *Perceived prosocial motivation moderates the positive indirect relationship between receiving autonomous help and task performance via task-focused processes, such that it is stronger with higher perceived prosocial motivation*.

**Hypothesis** **8** **(H8).** *Perceived prosocial motivation moderates the negative indirect relationship between receiving dependent help and task performance via self-focused processes, such that it is stronger with higher perceived prosocial motivation*.

**Figure 1:**
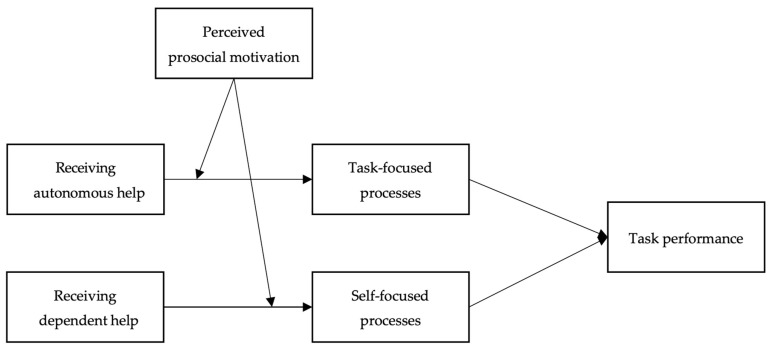
Theoretical Model.

Our hypothesized model is shown in Figure 1 as follows.

## 3. Method

### 3.1. Sample and Procedure

We conducted a two-wave, two-source survey among a convenience sample of full-time working adults. Participants were recruited through the researchers’ personal network and the alumni network of a university located in central China. Questionnaires were distributed and collected online. Before the survey started, we sent email invitations to potential employee participants explaining the study details (e.g., study purpose and procedure) and asking for their consent. We also asked the employee participants to help invite and obtain consent from their direct supervisors to take part in our survey. Only those who agreed to participate were sent links to online surveys. All participants were assured that their responses would be anonymous and confidential. We incentivized participants with CNY 30 (approximately USD 4) for their participation. In the first wave survey (T1), participants responded to questions on receiving autonomous and dependent help from coworkers, the perceived prosocial motivation of the coworkers that helped them, public self-consciousness, demographics, and contact information of their direct supervisors. We obtained responses from 365 employees. Two weeks later, the same 365 employees and their direct supervisors were invited to participate in the second wave survey (T2). Specifically, employees rated their self-focused processes and task-focused processes, and their direct supervisors rated their task performance. We finally obtained paired responses from 350 employees and supervisors, yielding a response rate of 95.89%. Of the 350 employees, 59.14% were female, and 79.71% held a bachelor’s degree or higher. Their average age was 30.63 years (ranging from 20 to 53 years), and their main organizational tenure was 3.91 years (ranging from less than 1 month to 22.08 years). Of the 350 supervisors, 46.29% were female, 94.86% held a bachelor’s degree or higher, their average age was 37.99 years (ranging from 25 to 55 years), and their average organizational tenure was 8.14 years (ranging from 7 months to 36.83 years).

### 3.2. Measures

All measures were rated on a 5-point Likert scale ranging from 1 (“strongly disagree”) to 5 (“strongly agree”). All measures used in this study were originally in English. We followed Brislin’s (1986) back-translation procedure when translating English into Chinese. Measures for all main variables can be found in our OSF repository: https://osf.io/rqhtc/?view_only=e512bca676504881b0faf36f3b7f20e6 (accessed on 27 November 2022).

**Receiving autonomous and dependent help.** We measured receiving autonomous and dependent help using the ten-item scale adapted from Koopmann [30]. We modified the original items following a referent-shift model by adapting “team members” and “our supervisor” to “I” and “my coworkers”, respectively. For example, we modified the original item, “When team members encounter work-task-related difficulties, our supervisor helps explain the substance of the issue so that we can solve the problem on our own in the future” to “When I encounter work-task-related difficulties, my coworkers help explain the substance of the issue so that I can solve the problem on my own in the future”. Participants were asked to rate the extent to which they agreed with the items, describing helping behaviors they received from coworkers during the past month. A sample item for receiving autonomous help was, “When I encounter work-task-related difficulties, my coworkers help explain the substance of the issue so that I can solve the problem on my own in the future”. A sample item for receiving dependent help was, “When I encounter work-task-related difficulties, my coworkers help me by handing me the solution”. The Cronbach’s alphas for receiving autonomous and dependent help were 0.93 and 0.78, respectively.

**Task-focused processes and self-focused processes.** We measured the task-focused processes and self-focused processes adapted from Kim and Kim [38]. Employees were asked to rate the extent to which they agreed with the items describing them when they were helped during the past month. The task-focused processes scale had four items (e.g., “My coworkers’ help made me pay more attention to how I conduct my tasks.”), and the self-focused processes scale had five items (e.g., “My coworkers’ help made me care about how I present myself to my colleagues.”). The Cronbach’s alphas for the task-focused processes and self-focused processes were 0.87 and 0.92, respectively.

**Perceived prosocial motivation.** Lanaj et al. [61] adapted three items by Grant [62] to measure the prosocial motivation of helping behavior. We modified the three items following a referent-shift model by adapting “I” to “my coworkers”. Employees were asked to rate the extent to which they agreed with the items describing the motivation of coworkers who helped them. A sample item is, “Because my coworkers want to have a positive impact on me through their help”. The Cronbach’s alpha for the scale was 0.94.

**Task performance.** We measured employee task performance using Methot et al.’s [63] five-item scale. Direct supervisors were asked to rate the extent to which they agree with the items describing “X” (a focal employee’s name inserted). A sample item is, “Adequately completes assigned duties”. The Cronbach’s alpha for the scale was 0.90.

**Control variables.** We controlled for employee age, gender (0 = female, 1 = male), educational level (1 = senior high or below; 2 = college or associate’s degree; 3 = bachelor’s degree; 4 = master’s degree; 5 = doctor’s degree or above), and organizational tenure (in years). Prior research suggests that these variables potentially influence employees’ experience of receiving help and performance [7,9,64,65]. We also controlled for public self-consciousness, which refers to the dispositional tendency to be aware of one’s appearance and concerned about making a good impression on others [66]. Previous research indicates that people with high public self-consciousness are more likely to attend to how they are perceived by others [67]. We measured public self-consciousness using Scheier and Carver’s [68] five-item scale. A sample item is, “I care a lot about how I present myself to others”. The Cronbach’s alpha for the scale was 0.77.

### 3.3. Analytical Strategy

We used Mplus 7.4 [69] to examine our hypotheses. We estimated two path models to test our hypotheses. In the indirect effect model, we considered the indirect relationships between the two types of coworker help and task performance via task-focused processes and self-focused processes. In the first-stage moderated indirect effect model, we additionally took the interaction terms into account. We grand-mean centered independent variables and the moderator to minimize potential multicollinearity problems. To test the indirect effects hypotheses, we followed the procedure recommended by MacKinnon, Fairchild, and Fritz [70] to calculate the confidence intervals (CI) for indirect effects using a bootstrap simulation with 5000 repetitions. To test the first-stage moderating effects, we calculated and plotted the interactions at conditional values of the moderators (1 *SD* above and below the mean) following Aiken and West’s [71] recommendation. To test the conditional indirect effects, we followed Preacher, Rucker, and Hayes [72] to calculate the indirect effects at high and low levels of the moderators and used the bootstrap simulation with 5000 repetitions to examine the results.

## 4. Results

### 4.1. Descriptive Statistics and Confirmatory Factor Analysis

A confirmatory factor analysis was conducted with the maximum-likelihood (ML) estimation using Mplus 7.4. The results indicated that our hypothesized six-factor model (receiving autonomous help, receiving dependent help, task-focused processes, self-focused processes, perceived prosocial motivation, and task performance) demonstrated an acceptable model fit to the data (χ^2^ (309) = 843.76, *p* < 0.001, SRMR = 0.08, RMSEA = 0.07, CFI = 0.92, TLI = 0.91) and was better than a five-factor model that combined receiving autonomous help and dependent help (χ^2^ (314) = 1220.74, $\Delta \chi^{2}\;$(5) = 376.98, *p* < 0.001, CFI = 0.86, TLI = 0.84, RMSEA = 0.09, SRMR = 0.08); a five-factor model that combined task-focused processes and self-focused processes (χ^2^ (314) = 1587.18, $\Delta \chi^{2}\;$(5) = 743.42, *p* < 0.001, CFI = 0.80, TLI = 0.78, RMSEA = 0.11, SRMR= 0.14); and a three-factor model that combined receiving autonomous help, receiving dependent help, task-focused processes, and self-focused processes (χ^2^ (321) = 2996.69, $\Delta \chi^{2}\;$(12) = 2152.93, *p* < 0.001, CFI = 0.58, TLI = 0.54, RMSEA = 0.15, SRMR = 0.15). These results supported the discriminant validity of our focal variables.

Table 2 reports the means, standard deviations, reliability coefficients, and correlations of the variables in the study. The alpha reliabilities for all scales were greater than 0.77. Bivariate correlations showed that receiving autonomous help was positively related to task-focused processes (r = 0.34; *p* < 0.01); receiving dependent help was positively related to self-focused processes (r = 0.14; *p* < 0.01); and task-focused processes (r = 0.18; *p* < 0.01) were positively related to task performance, whereas self-focused processes (r = −0.13; *p* < 0.05) were negatively related to task performance.

### 4.2. Test of the Mediating Effects

We tested Hypotheses 1, 2, 3, and 4 using an indirect effect model in which receiving autonomous and dependent help served as the independent variables, task-focused and self-focused processes served as the mediators, and task performance served as the dependent variable. As shown in Table 3, receiving autonomous help was positively related to task-focused processes (B = 0.17, SE = 0.07, *p* = 0.01), receiving dependent help was positively related to self-focused processes (B = 0.26, SE = 0.08, *p* = 0.00), task-focused processes were positively related to task performance (B = 0.13, SE = 0.06, *p* = 0.02), and self-focused processes were negatively related to task performance (B = −0.12, SE = 0.05, *p* = 0.01). We further conducted bootstrap simulation with 5000 repetitions to examine the mediating effects. Results shown in Table 4 suggest that the indirect relationship between receiving autonomous help and task performance via task-focused processes was 0.02 (95% CI = [0.00, 0.06]); and the indirect relationship between receiving dependent help and task performance via self-focused processes was −0.03 (95% CI = [−0.07, −0.01]). Therefore, Hypotheses 1, 2, 3, and 4 were supported.

### 4.3. Test of the Moderating Effects

**Figure 2:**
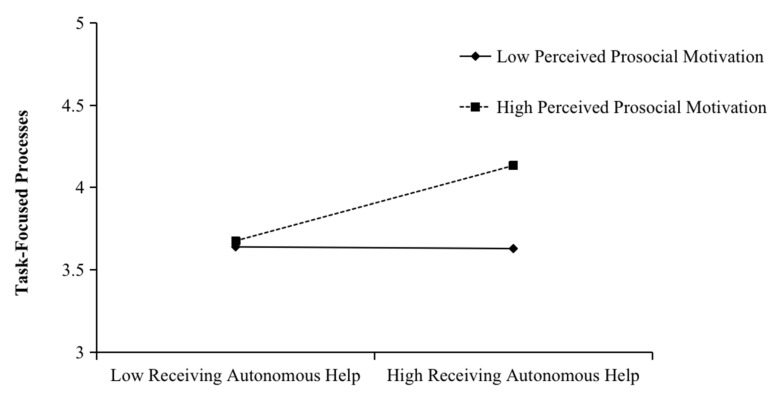
Moderating effect of perceived prosocial motivation on the relationship between receiving autonomous help and task-focused processes.

**Figure 3:**
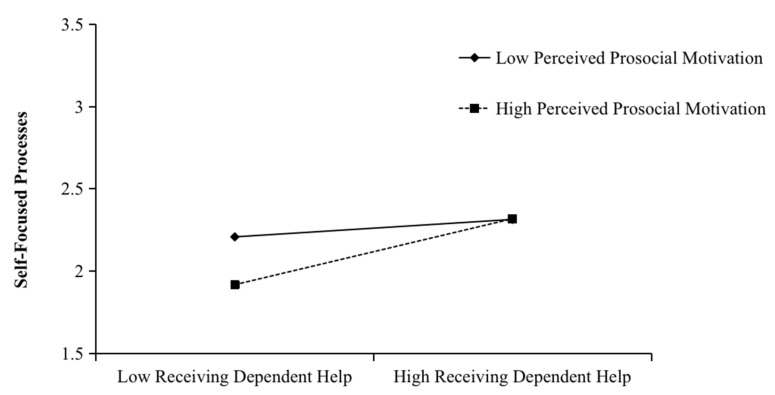
Moderating effect of perceived prosocial motivation on the relationship between receiving dependent help and self-focused processes.

We tested Hypotheses 5, 6, 7, and 8 using a first-stage moderated indirect model on the indirect effect model by adding the interaction terms between receiving autonomous help and perceived prosocial motivation, and receiving dependent help and perceived prosocial motivation. As shown in Table 3, the interaction between receiving autonomous help and perceived prosocial motivation was positively related to task-focused processes (B = 0.24, SE = 0.08, *p* = 0.00). The interaction between receiving dependent help and perceived prosocial motivation was positively related to self-focused processes (B = 0.17, SE = 0.08, *p* = 0.04). We then plotted the interaction at conditional values of perceived prosocial motivation (1 SD above and below the mean). As shown in Figure 2, receiving autonomous help was more positively related to task-focused processes when perceived prosocial motivation was high (B = 0.34, SE = 0.08, *p* = 0.00) than when it was low (B = −0.01, SE = 0.10, *p* = 0.94), and the difference was significant (difference = 0.35, SE = 0.12, *p* = 0.00). As shown in Figure 3, receiving dependent help was more positively related to self-focused processes when perceived prosocial motivation was high (B = 0.32, SE = 0.08, *p* = 0.00) than when it was low (B = 0.08, SE = 0.12, *p* = 0.46), and the difference was significant (difference = 0.24, SE = 0.12, *p* = 0.04). Thus, Hypotheses 5 and 6 were supported.

Next, we computed the confidence intervals for the conditional indirect effects at high and low values of perceived prosocial motivation using bootstrap simulation (5000 repetitions). Results shown in Table 5 indicate that receiving autonomous help was positively related to task performance via task-focused processes when perceived prosocial motivation was high (indirect effect = 0.04, 95% CI = [0.01, 0.09]); the indirect relationship was nonsignificant when perceived prosocial motivation was low (indirect effect = −0.00, 95% CI = [−0.03, 0.03]); and the difference between the two was significant (difference = 0.040, 95% CI = [0.01, 0.11]). Receiving dependent help was negatively related to task performance via self-focused processes when perceived prosocial motivation was high (indirect effect = −0.04, 95% CI = [−0.08, −0.01]), and the indirect relationship was nonsignificant when perceived prosocial motivation was low (indirect effect = −0.01, 95% CI = [−0.05, 0.01]). The difference between the two was significant (difference = −0.03, 95% CI = [−0.08, −0.00]). Thus, Hypotheses 7 and 8 were supported.

We obtained the same pattern of results without control variables.

## 5. General Discussion

In this study, we examined the differential effects of receiving autonomous and dependent help on recipient processing modes and subsequent task performance and uncovered a boundary for these effects. The results based on two-wave and two-source data showed that receiving autonomous help had a positive indirect relationship with task performance by fostering task-focused processes. In contrast, receiving dependent help had a negative indirect relationship with task performance by diverting recipients to self-focused processes. In addition, our results revealed that perceived prosocial motivation augmented the effects of receiving autonomous and dependent help on the respective processing mode and subsequent task performance.

### 5.1. Theoretical Contributions

Our research makes several contributions to the helping literature. First, this study extends the understanding of the differential influence of receiving autonomous help and dependent help on recipient task performance. Despite the prevailing consensus that autonomous help benefits recipients more than dependent help [4,8,29,30], few studies have examined the specific mechanisms through which the two types of help impact recipient task performance (except for [7,9,10]). Particularly, existing research has primarily focused on the role of recipients’ perceived competence in explaining the effects of the two types of help on work outcomes. By introducing two opposing processing modes, task-focused and self-focused processes, our study complements the literature by providing an integrative framework that further delineates how receiving autonomous help and dependent help can impose divergent impacts on task performance.

Second, our research adds a new theoretical perspective by highlighting the value of the informational function of coworker help. Earlier helping research suggested that help may contain highly threatening messages that emphasize one’s relative dependency and inferiority to peers, or it may contain more supportive information, allowing the recipients to feel as skilled as others [73,74]. Integrating these theoretical cues into social information processing mechanisms, we propose that receiving autonomous help directs recipients’ attention to task-focused processes by conveying developmental information. In contrast, dependent help delivers evaluative information that directs recipients’ attention to self-focused processes. In so doing, our study contributes to understanding how coworker help serves as important social information in the workplace that affects recipients’ responses.

Third, our findings suggest that the effects of receiving coworker help are influenced not merely by the characteristics of helping actions but also by the attribution of helping motivation of the helpers. In so doing, we expand the boundary conditions of our mediating mechanism, providing a new way to reconcile the conflicting views regarding the role of help in recipients’ task performance. Furthermore, recent findings in the helping and sexism literature indicate that benevolent intentions may cause subtle negative consequences [64,75]. Our research extends this line of work by showing that, under high perceived prosocial motivation, the receipt of dependent help is more likely to trigger intrusive thoughts, cause recipients to be caught in self-focused processes, and thus decrease task performance. Thus, our research also contributes to understanding the potential negative effects of perceived prosocial motivation.

### 5.2. Practical Implications

This research provides several practical implications for helpers and managers. First, helpers should be aware that even though help is well-intentioned, it can still have potentially harmful effects on recipients, especially when given as dependent help. Our results demonstrate the advantages of receiving autonomous help in improving task-focused processes and task performance. Therefore, helpers need to be attentive to the way in which they provide help. For example, they can share their personal experiences, suggest different perspectives, and help coworkers identify ways to find solutions rather than giving them solutions.

In addition, managers should create a conducive climate that encourages employees to provide each other with autonomous help. Specifically, communicate learning-oriented beliefs and values to subordinates and provide incentives for them to participate in autonomous help. Notably, sometimes dependent help is necessary, such as when recipients face excessive workloads or role conflict. Managers should be aware of how to avoid the potential negative effects of receiving dependent help on employees. For example, they could communicate with employees about the negative beliefs employees may have when receiving dependent help and let employees realize that they can proactively request a detailed explanation of the solution after receiving dependent help in order to gain relevant knowledge and skills.

### 5.3. Limitations and Future Directions

Several limitations of our research should be noted. First, although we collected multi-time data and multi-sourced data, we cannot make causal inferences or rule out reverse causality due to the survey design. One potential is that higher-performing recipients are more likely to be provided with autonomous help, whereas lower-performing recipients are more likely to be provided with dependent help. To address this issue, we suggest future studies to use experimental or cross-lagged panel designs to substantiate the causality of reciprocal relationships.

Second, we focused on perceived prosocial motivation to examine the moderating effect of motivational attribution on recipients’ processing mechanisms after receiving help. However, we recognize that there are other motivational attributions for helping behavior, such as perceived impression management motivation [25,49,74]. We therefore encourage future research to explore the moderating role of other motivational attributes for help. In addition, recipients’ needs may be closely related to subsequent responses, for example, whether the help is in need or whether the type of help received matches the type of help sought. We encourage future research to explore other boundary conditions.

Finally, all participants in our sample are from China; thus, cultural factors might influence our findings. Chinese culture values interpersonal relationships and expects interdependence among ‘insiders’ [76] (pp. 35–39), [77]. It is reasonable to assume that interpersonal closeness between helper and recipient might decrease the negative experience of receiving dependent help such as self-focused processes. Therefore, we suggest future studies collect dyadic data and examine the moderating effect of relationship closeness. In addition, since we use convenience samples in the current study, the generalizability of our findings will be limited. We therefore encourage researchers to replicate our findings using more representative samples and samples across different cultures.

## 6. Conclusions

The distinction between receiving autonomous and dependent help from the social information processing perspective further emphasizes the idea that the experience of receiving help is a double-edged sword. We find a positive indirect relationship between coworker autonomous help and recipient task performance through enhanced task-focused processes. By contrast, there is a negative indirect relationship between coworker dependent help and recipient task performance through enhanced self-focused processes. Moreover, perceived prosocial motivation aggravates the effects of coworker autonomous and dependent help on respective processing modes and subsequent task performance. We hope the current study will stimulate future research advancing our understanding of the differential impacts of coworker autonomous and dependent help and examining other contingencies for the effects of the two types of help on task performance.

## Figures and Tables

**Table 1:** A Summary of Research on Workplace Autonomous and Dependent Help.

Authors, Date	Research Perspective	Main Findings Regarding Consequences on Help Recipients/Seekers
Geller & Bamberger [4]	Help-seeking	Help-seeking is more strongly related to performance when autonomous help-seeking logic is high, and more weakly related to performance when dependent help-seeking logic is high.
Komissarouk et al. [29]	Help-seeking	Preference for autonomous help-seeking leads to higher performance ratings by supervisors.
Koopmann [30]	Receiving help	Leader autonomous and dependent help were positively related to team autonomous and dependent help, respectively. Team autonomous help increased team job satisfaction.
Lee et al. [7]	Receiving help	Receiving empowering (autonomous) help increased perception of competence; receiving non-empowering (dependent) help increased perceived competence only for male recipients; perception of competence further transmitted such effects to increased work-goal progress, enacted task-focused helping, and decreased withdrawal.
Lin [8]	Receiving help	Receipt of autonomous help enhanced employees’ self-efficacy and feeling of gratitude; receipt of dependent help decreased employees’ self-efficacy and feeling of gratitude, and such effects were stronger when autonomous help was lacking. Furthermore, feelings of gratitude, rather than self-efficacy, mediated the effects of receiving help on employees’ performance and psychological well-being outcomes.
Liu et al. [9]	Help-seeking	Autonomous help-seeking facilitated help-seekers’ job performance through self-perceived competence; dependent help-seeking hampered their job performance through coworker-perceived competence.
Zhu et al. [10]	Receiving help	Autonomous help from leaders had a positive effect on work role performance of members via self-efficacy, and the leader–member exchange relationship further strengthened such effect.

**Table 2:** Means, Standard Deviations, and Correlations among Variables.

Variable	Mean	SD	1	2	3	4	5	6	7	8	9	10	11
1. Receiving autonomous help (T1)	3.44	0.68	(0.93)										
2. Receiving dependent help (T1)	2.92	0.63	0.23 **	(0.78)									
3. Task-focused processes (T2)	3.71	0.65	0.34 **	0.13 *	(0.87)								
4. Self-focused processes (T2)	2.91	0.83	−0.10	0.14 **	0.05	(0.92)							
5. Perceived prosocial motivation (T1)	3.45	0.71	0.67 **	0.14 **	0.35 **	−0.11 *	(0.94)						
6. Task performance (T2)	3.65	0.64	0.19 **	0.04	0.18 **	−0.13 *	0.22 **	(0.90)					
7. Age	30.63	6.04	0.09	0.08	0.08	−0.05	0.08	0.06	—				
8. Gender	0.41	0.49	−0.06	0.14 **	−0.09	−0.07	−0.08	0.03	−0.09	—			
9. Educational level	2.95	0.81	−0.07	−0.09	−0.06	0.04	−0.07	−0.04	−0.38 **	0.02	—		
10. Tenure	3.91	3.09	−0.04	−0.05	0.05	0.05	−0.04	0.05	0.68 **	−0.07	−0.17 **	—	
11. Public self-consciousness (T1)	2.74	0.63	−0.19 **	0.00	−0.08	0.36 **	−0.08	0.00	−0.03	−0.02	0.00	0.00	(0.77)

*Notes*: *N* = 350. Reliabilities along the diagonal in parentheses. * *p* < 0.05, ** *p* < 0.01 (two tailed).

**Table 3:** Hypotheses Testing Results.

Variable	Model 1						Model 2					
	Task-Focused Processes		Self-Focused Processes		Task Performance		Task-Focused Processes		Self-Focused Processes		Task Performance	
	B	SE	B	SE	B	SE	B	SE	B	SE	B	SE
Intercept	2.39 **	0.45	1.83 **	0.51	2.66 **	0.40	3.77 **	0.32	2.19 **	0.43	3.31 **	0.38
Age	−0.00	0.01	−0.02 *	0.01	−0.00	0.01	−0.00	0.01	−0.02	0.01	−0.00	0.01
Gender	−0.08	0.07	−0.17 *	0.09	0.06	0.07	−0.10	0.07	−0.17 *	0.08	0.06	0.07
Educational level	−0.001	0.04	0.02	0.06	−0.01	0.05	0.02	0.04	0.01	0.06	−0.01	0.05
Tenure	0.02	0.02	0.04 *	0.02	0.02	0.01	0.02	0.01	0.04 *	0.02	0.02	0.01
PSC	−0.03	0.06	0.46 **	0.07	0.09	0.06	−0.03	0.06	0.44 **	0.07	0.09	0.06
AH	0.17 *	0.07	0.00	0.09	0.07	0.07	0.17 *	0.07	−0.00	0.09	0.07	0.07
DH	0.07	0.06	0.26 **	0.08	0.01	0.06	0.12	0.07	0.20 *	0.08	0.02	0.06
PPM	0.20 **	0.07	−0.12	0.08	0.11	0.06	0.19 **	0.06	−0.10	0.08	0.11	0.06
AH * PPM							0.24 **	0.08	−0.17 *	0.08	0.02	0.07
DH * PPM							−0.09	0.08	0.17 *	0.08	−0.02	0.07
Task-focused processes					0.13 *	0.06					0.12 *	0.06
Self-focused processes					−0.12 *	0.05					−0.12 *	0.05
*R^2^*	0.16 **		0.19 **		0.09 **		0.21 **		0.20 **		0.09 **	

*Notes*: *N* = 350. Table entries report unstandardized parameter estimates with standard errors. Abbreviations: AH = receiving autonomous help; DH = receiving dependent help; PPM = perceived prosocial motivation; PSC = public self-consciousness. * *p* < 0.05, ** *p* < 0.01 (two tailed).

**Table 4:** Indirect Effect Results.

Mediation Effect	Estimates	95% CI
Receiving autonomous help–Task-focused processes–Task performance	0.02	[0.00 0.06]
Receiving dependent help–Self-focused processes–Task performance	−0.03	[−0.07 −0.01]

*Notes*: CI refers to bootstrapped confidence intervals. Replications = 5000.

**Table 5:** Conditional Indirect Effect Results.

	Estimates	95% CI
*Receiving autonomous help–Task-focused processes–Task performance*		
Low perceived prosocial motivation	−0.00	[−0.03 0.03]
High perceived prosocial motivation	0.04	[0.01 0.09]
Difference	0.04	[0.01 0.11]
*Receiving dependent help–Self-focused processes–Task performance*		
Low perceived prosocial motivation	−0.01	[−0.05 0.01]
High perceived prosocial motivation	−0.04	[−0.08 −0.01]
Difference	−0.03	[−0.08 −0.00]

*Notes:* CI refers to bootstrapped confidence intervals. Replications = 5000.

## Data Availability

Not applicable.
